# Benchmark on a large cohort for sleep-wake classification with machine learning techniques

**DOI:** 10.1038/s41746-019-0126-9

**Published:** 2019-06-07

**Authors:** Joao Palotti, Raghvendra Mall, Michael Aupetit, Michael Rueschman, Meghna Singh, Aarti Sathyanarayana, Shahrad Taheri, Luis Fernandez-Luque

**Affiliations:** 1 Qatar Computing Research Institute, HBKU, Doha, Qatar; 2 Brigham and Women’s Hospital, Boston, MA USA; 3 Harvard University, Boston, MA USA; 4 University of Minnesota, Minneapolis, MN USA; 5 Boston Children’s Hospital, Boston, MA USA; 6 Weill Cornell Medicine Qatar, Doha, Qatar

**Keywords:** Machine learning, Health care, Epidemiology, Quality of life, Fatigue

## Abstract

Accurately measuring sleep and its quality with polysomnography (PSG) is an expensive task. Actigraphy, an alternative, has been proven cheap and relatively accurate. However, the largest experiments conducted to date, have had only hundreds of participants. In this work, we processed the data of the recently published Multi-Ethnic Study of Atherosclerosis (MESA) Sleep study to have both PSG and actigraphy data synchronized. We propose the adoption of this publicly available large dataset, which is at least one order of magnitude larger than any other dataset, to systematically compare existing methods for the detection of sleep-wake stages, thus fostering the creation of new algorithms. We also implemented and compared state-of-the-art methods to score sleep-wake stages, which range from the widely used traditional algorithms to recent machine learning approaches. We identified among the traditional algorithms, two approaches that perform better than the algorithm implemented by the actigraphy device used in the MESA Sleep experiments. The performance, in regards to accuracy and *F*_1_ score of the machine learning algorithms, was also superior to the device’s native algorithm and comparable to human annotation. Future research in developing new sleep-wake scoring algorithms, in particular, machine learning approaches, will be highly facilitated by the cohort used here. We exemplify this potential by showing that two particular deep-learning architectures, CNN and LSTM, among the many recently created, can achieve accuracy scores significantly higher than other methods for the same tasks.

## Introduction

Short and poor quality sleep have been directly linked to a series of chronic health problems, including obesity, insulin resistance, and hypertension.^1–4^ Thus, measuring sleep and its quality are increasingly important beyond the diagnosis of specific sleep disorders.

While polysomnography (PSG) is the gold standard approach for diagnosing specific sleep disorders, it is impractical for use in the identification of more prevalent issues with sleep loss and sleep quality. An attractive alternative to PSG is the use of wearables, such as accelerometer-based technology (Actigraphy), which may be used as a diagnostic aid for specific sleep disorders such as circadian rhythm disorders.

Actigraphy devices allow several weeks of unobtrusive, continuous recording, enabling prospective, and naturalistic assessment of sleep.^5^ While the signals captured by an actigraphy device are not as detailed as the ones obtained by PSG, it allows the identification of sleep-wake states, sleep timing, and sleep quality.^5^

Over the past three decades, a number of studies have demonstrated the reliability and validity of actigraphy to replace PSG for nocturnal sleep-wake scoring.^5–13^ These studies show an epoch-by-epoch agreement between activity-based sleep-wake scoring algorithms and traditional PSG-based scoring ranging between 80 and 95%. This accuracy helped in making the usage of actigraphy devices a part of sleep medicine guidelines for the diagnosis of a number of sleep disorders.^14^

Nevertheless, an existing challenge for actigraphy studies is the relative difficulty in comparing the performance of different actigraphy algorithms due to the lack of standardized datasets.^15^ Although recent studies have assessed the validity of scoring algorithms in comparison with PSG,^8,16^ they are usually made with a small number of participants due to the complexity of conducting these studies.

Until very recently, one of the main barriers for the development and enhancement of artificial intelligence methods in sleep research was the lack of public repositories of actigraphy data and tools. However, that trend is changing with recent initiatives, such as sleepdata.org from the National Sleep Research Resource (NSRR), which allows researchers to freely access large collections of well-characterized research cohorts and clinical trials.^17,18^ One such dataset is the Multi-Ethnic Study of Atherosclerosis (MESA).

MESA was a research study investigating factors associated with the development of subclinical cardiovascular disease and the progression of subclinical to clinical cardiovascular disease in 6814 individuals. The participants were men and women between 45 and 84 years of age at the beginning of the study, from different ethnic communities (Black, White, Hispanic, and Chinese-American). Between 2010 and 2012, approximately one-third of the participants (2237) were enrolled for sleep assessment (MESA Sleep), which included one full overnight unattended PSG session, 7-day wrist-worn actigraphy, and a sleep questionnaire.

In this work, we propose to use the MESA dataset as a cohort to compare the performance of existing and future sleep-wake scoring algorithms. We leveraged the fact that the MESA Sleep dataset is the largest dataset to date for studying actigraphy-based sleep-wake scoring algorithms, being a hundred times bigger than previously used datasets. The Supplementary Table 1 summarizes the basic statistics of the part of MESA Sleep dataset used in this work and compares it to the related work.

The contribution of this work is threefold:

- First, we build a standardized benchmark to serve the development of new ideas and approaches. We propose two specific research tasks for this cohort: Task Night and Task Night&Day.
- Second, we review, investigate and validate the main heuristics to identify wake-sleep patterns from actigraphy devices. In our study, we include both well-established heuristics and algorithms, and new state-of-the-art machine learning algorithms. We aim to foster future artificial intelligence research into sleep medicine, and the methods described here will serve as baselines for future research.
- Third, we make available to the community, a Python library for sleep-wake scoring with all algorithms implemented in this paper (and tools to facilitate the implementation of new algorithms in the future). The code and data used can be found online at https://github.com/qcri/sleep_awake_benchmark.

## Results

The performance of machine learning methods is influenced by the choices of optimal hyperparameters. The only hyperparameter optimized for the traditional scoring formulas was *Oakley’s* threshold *θ*, which was set to 10, the value that yielded the highest accuracy score in the training set. We show results for *θ* = 40 and *θ* = 80 as well, as these values are commonly used in the literature.^16^ In particular, *θ* = 40 is the device algorithm.^19,20^ Hyperparameters of ML and DL algorithms were obtained via the standard fivefold cross-validation while optimizing for accuracy. A detailed list of all the hyperparameters for each model that we explored in this work are provided in the Supplementary Material.

### Task night results: predicting sleep quality metrics during night

The results of the experiments of Task Night are shown in Table 1. We group the results according to the technique used (traditional algorithms, ML algorithms and DL algorithms), and whether Webster rescoring rules were used or not. Within each group, we sort the results by mean accuracy in descending order.

**Table 1:** Results (Mean ± 95% confidence interval) for Task Night

Method	Algorithm evaluation metrics					Sleep quality metrics			
	Accuracy	Specificity	Precision	Sensitivity	F1	WASO (min)	MAE WASO	Sleep Eff. (%)	MAE sleep Eff.
Ground truth	100.0 ± 0.0	100.0 ± 0.0	100.0 ± 0.0	100.0 ± 0.0	100.0 ± 0.0	102.1 ± 7.3	0.0	58.4 ± 1.4	0.0
Baselines									
Manual annotations	**79.8** ± **1.2**	56.5 ± 2.3	**75.8** ± **1.5**	94.8 ± 1.5	**83.3** ± **1.4**	45.8 ± 8.6	74.7	**73.0** ± **1.7**	**17.2**
Device algorithm	76.2 ± 1.0	50.1 ± 1.8	72.6 ± 1.3	94.3 ± 0.6	81.3 ± 1.0	**54.0** ± **4.2**	**53.1**	75.7 ± 1.0	17.7
Always sleep	58.4 ± 1.4	0.0 ± 0.0	58.4 ± 1.4	**100.0** ± **0.0**	72.8 ± 1.1	0.0 ± 0.0	102.1	100.0 ± 0.0	41.6
Always wake	41.6 ± 1.4	**100.0** ± **0.0**	0.0 ± 0.0	0.0 ± 0.0	0.0 ± 0.0	459.2 ± 9.0	357.0	0.0 ± 0.0	58.4
Traditional algorithms									
Oakley * θ*= 10^32^	**77.5** ± **0.9**	63.0 ± 1.7	76.8 ± 1.3	87.2 ± 0.9	81.0 ± 1.0	**95.0**± **5.9**	**37.3**	66.0 ± 1.1	10.1
Scripps Clinic^21^	76.6 ± 1.1	48.8 ± 1.9	72.5 ± 1.4	95.9 ± 0.5	**81.8** ± **1.0**	46.3 ± 4.2	58.5	77.1 ± 1.0	18.9
Oakley * θ*= 40^32^	75.9 ± 1.0	49.3 ± 1.8	72.2 ± 1.3	94.4 ± 0.5	81.2 ± 1.0	53.1 ± 4.1	52.9	76.0 ± 1.0	17.9
Cole-Kripke^6^	75.4 ± 1.1	45.0 ± 1.8	71.1 ± 1.4	96.7 ± 0.4	81.2 ± 1.0	40.2 ± 3.7	63.5	79.2 ± 1.0	21.0
Sazonov^9^	75.2 ± 1.0	**73.3** ± **1.6**	**79.9** ± **1.3**	75.5 ± 1.4	76.7 ± 1.2	149.2 ± 7.7	58.7	**54.9** ± **1.3**	**9.1**
Oakley * θ* = 80^32^	73.9 ± 1.1	41.2 ± 1.7	69.7 ± 1.4	96.9 ± 0.4	80.3 ± 1.0	35.9 ± 3.2	67.4	80.9 ± 0.9	22.7
Sadeh^5^	73.4 ± 1.2	38.3 ± 1.8	69.1 ± 1.4	**98.3** ± **0.3**	80.3 ± 1.1	26.3 ± 3.1	76.5	83.0 ± 0.9	24.7
Webster^28^	73.3 ± 1.2	38.2 ± 1.8	69.0 ± 1.4	98.2 ± 0.3	80.3 ± 1.1	27.5 ± 3.0	75.3	83.0 ± 0.9	24.7
Group average	75.1 ± 1.3	49.6 ± 10.4	72.5 ± 3.3	92.9 ± 6.6	80.4 ± 1.3	59.2 ± 35.4	61.3 ± 10.6	75.0 ± 8.2	18.6 ± 5.1
Rescoring rules applied to traditional algorithms									
Resc. Oakley * θ* = 40	**80.3** ± **0.9**	68.3 ± 1.9	79.9 ± 1.3	88.1 ± 0.9	83.1 ± 1.0	93.2 ± 6.6	**37.7**	64.4 ± 1.2	**9.0**
Resc. Cole-Kripke	80.2 ± 1.0	65.7 ± 2.0	78.9 ± 1.3	89.9 ±0.8	83.3 ± 1.0	83.5 ± 6.3	40.0	66.6 ± 1.2	10.2
Resc. Scripps Clinic	80.1 ± 1.0	70.4 ± 1.9	80.7 ± 1.3	86.3 ± 1.1	82.6 ± 1.0	**102.8** ± **7.5**	41.8	**62.5** ± **1.3**	9.1
Resc. Oakley * θ* = 80	79.3 ± 1.0	59.8 ± 2.0	76.6 ± 1.4	92.8 ± 0.6	**83.2** ± **1.0**	65.0 ± 5.4	46.4	70.7 ± 1.1	13.1
Resc. Sadeh	79.1 ± 1.0	59.4 ± 2.0	76.5 ± 1.4	**92.8** ± **0.7**	83.1 ± 1.0	64.1 ± 5.7	49.2	70.9 ± 1.2	13.5
Resc. Webster	79.0 ± 1.0	58.9 ± 2.0	76.2 ± 1.4	93.1 ± 0.7	83.0 ± 1.0	63.2 ± 5.5	48.9	71.3 ± 1.2	13.8
Resc. Oakley * θ* = 10	77.8 ± 1.0	81.6 ± 1.6	85.5 ± 1.3	73.8 ± 1.6	78.0 ± 1.3	163.9 ± 9.1	68.7	50.7 ± 1.5	10.8
Resc. Sazonov	68.1 ± 1.3	**90.1** ± **1.3**	**87.8** ± **1.6**	51.2 ± 2.1	62.3 ±2.0	258.4 ± 11.0	156.7	34.0 ± 1.6	24.7
Group average	78.0 ± 3.4	69.3 ± 9.5	80.3 ± 3.6	83.5 ± 12.1	79.8 ± 6.1	111.8 ± 56.8	61.2 ± 33.3	61.4 ± 10.9	13.0 ± 4.3
Machine learning algorithms									
Extra trees	**81.8** ± **1.0**	68.1 ± 1.9	**80.3** ± **1.3**	90.4 ± 1.2	**84.3** ± **1.1**	85.4 ± 7.4	**42.8**	65.8 ± 1.4	10.3
Logistic regression	81.5 ± 1.0	67.2 ± 2.0	79.9 ± 1.3	**90.7** ± **1.2**	84.1 ± 1.1	83.2 ± 7.5	45.6	66.3 ± 1.4	11.1
Linear SVM	81.4 ± 1.1	68.0 ± 2.0	80.2 ± 1.3	89.9 ± 1.3	83.8 ± 1.1	87.2 ± 7.8	45.8	65.5 ± 1.5	10.8
Perceptron	78.4 ± 1.0	**69.0** ± **1.8**	79.4 ± 1.3	83.9 ± 1.4	80.7 ± 1.2	**110.3** ± **8.0**	44.0	**61.7** ± **1.4**	**9.3**
Group average	80.8 ± 2.5	68.1 ± 1.2	80.0 ± 0.6	88.8 ± 5.1	83.2 ± 2.7	91.5 ± 20.1	44.6 ± 2.3	64.8 ± 3.4	10.4 ± 1.2
Rescoring rules applied to machine learning algorithms									
Resc. Log. Regression	**78.9** ± **1.2**	80.7 ± 1.8	85.6 ± 1.2	**75.9** ± **1.9**	**78.8** ± **1.5**	**152.8** ± **10.4**	**64.5**	**52.2** ± **1.7**	**10.6**
Resc. extra trees	78.5 ± 1.2	82.0 ± 1.7	**86.1** ± **1.2**	74.2 ± 1.9	78.2 ± 1.5	160.4 ± 10.3	68.6	50.8 ± 1.7	11.0
Resc. linear SVM	78.3 ± 1.2	81.4 ± 1.7	85.8 ± 1.2	74.4 ± 2.0	77.9 ± 1.6	159.4 ± 10.6	69.6	51.1 ± 1.7	11.2
Resc. perceptron	73.4 ± 1.3	**84.4** ± **1.5**	85.7 ± 1.4	63.8 ± 2.2	70.8 ± 1.9	202.2 ± 11.3	104.4	43.7 ± 1.8	16.2
Group average	77.3 ± 4.1	82.1 ± 2.6	86.0 ± 0.3	72.1 ± 8.9	76.4 ± 6.0	168.7 ± 35.9	76.8 ± 29.5	49.5 ± 6.2	12.2 ± 4.2
Deep-learning algorithms									
LSTM 100	**83.1** ± **1.0**	69.9 ± 2.0	**81.6** ± **1.3**	91.4 ± 1.1	**85.5** ± **1.0**	79.2 ± 7.6	43.9	65.6 ± 1.4	10.0
CNN 100	82.9 ± 1.0	68.8 ± 2.1	81.3 ± 1.3	91.7 ± 1.2	85.3 ± 1.1	78.3 ± 7.9	46.7	66.2 ± 1.5	10.8
LSTM 50	82.7 ± 1.0	**70.1** ± **1.9**	81.5 ± 1.3	90.5 ± 1.1	85.0 ± 1.0	**85.6** ± **7.6**	**41.3**	**64.9** ± **1.4**	**9.6**
CNN 50	82.5 ± 1.0	67.6 ± 2.0	80.5 ± 1.3	**92.0** ± **1.1**	85.1 ± 1.1	75.9 ± 7.4	46.6	66.9 ± 1.4	11.0
CNN 20	81.4 ± 1.0	66.5 ± 1.9	79.6 ± 1.3	90.9 ± 1.1	84.1 ± 1.1	81.9 ± 7.1	43.2	66.7 ± 1.4	10.8
LSTM 20	81.3 ± 1.0	65.0 ± 1.9	79.0 ± 1.3	92.0 ± 1.0	84.3 ± 1.0	75.3 ± 6.7	44.5	68.0 ± 1.3	11.4
Group average	82.3 ± 0.8	68.0 ± 2.1	80.6 ± 1.2	91.4 ± 0.7	84.9 ± 0.6	79.4 ± 4.1	44.4 ± 2.2	66.4 ± 1.1	10.6 ± 0.7
Rescoring rules applied to deep-learning algorithms									
Resc. LSTM 100	**81.2** ± **1.0**	77.8 ± 1.8	84.8 ± 1.2	**82.1** ± **1.5**	**82.3** ± **1.2**	**123.4** ± **9.4**	**47.2**	**57.1** ± **1.6**	**8.7**
Resc. CNN 100	80.9 ± 1.0	78.3 ± 1.9	85.1 ± 1.2	81.1 ± 1.7	81.7 ± 1.3	128.1 ± 9.9	50.8	56.4 ± 1.7	9.3
Resc. CNN 50	80.6 ± 1.1	78.2 ± 1.8	84.8 ± 1.3	80.6 ± 1.7	81.4 ±1.3	130.0 ± 9.7	51.4	56.1 ± 1.6	9.3
Resc. LSTM 50	79.9 ± 1.0	80.1 ± 1.7	85.6 ± 1.2	78.0 ± 1.6	80.4 ± 1.3	142.9 ± 9.8	55.6	53.8 ± 1.6	9.5
Resc. LSTM 20	79.5 ± 1.1	79.9 ± 1.7	85.2 ± 1.2	77.5 ± 1.7	79.9 ± 1.4	145.2 ± 9.8	56.9	53.6 ± 1.6	9.6
Resc. CNN 20	78.4 ± 1.1	**81.3** ± **1.7**	**85.7** ± **1.3**	74.5 ± 1.8	78.2 ± 1.5	158.5 ± 10.2	66.8	51.3 ± 1.7	10.8
Group average	80.1 ± 1.1	79.3 ± 1.5	85.2 ± 0.4	79.0 ± 3.0	80.7 ± 1.6	138.0 ± 13.8	54.8 ± 7.2	54.7 ± 2.3	9.6 ± 0.7

Methods within each group are sorted by their mean accuracy score. The best results for each category are marked in bold. Note that for WASO and Sleep Efficiency, the best results are the closest to the ground truth

Results of the baseline approaches *Always Sleep* and *Always Wake* show that 58.4% of the epochs in Task Night dataset are sleep and thus the minimum accuracy score that we should expect is 58.4. The proprietary *Device Algorithm* and the *Manual Annotation* have, respectively, an accuracy of 76.2 and 79.8, in line with other traditional algorithms, which vary from 73.3 (*Webster*) to 77.5 (*Oakley*_*θ*=10_). Note that this accuracy range is lower than the reported accuracy of 80–95% in original papers that introduced new algorithms (upper part of Table S1, e.g., refs ^6,21^), but it is similar to the reported 70–85% range of validation papers (lower part of Table S1, e.g., refs ^11,22,23^). Note that both *Manual Annotation* and *Device Algorithm* underestimate the number of wake epochs, resulting in the overestimation of sleep efficiency when compared to the *Oracle*. Also note that, as expected, *Oakley*_*θ*=40_ and *Device Algorithm* present very similar results, with no significant differences between the results of these two approaches.

Apart from *Sazonov*, all other traditional algorithms have a high sensitivity score (as high as 98.3 for *Sadeh* algorithm), but relatively smaller average precision score (highest is *Oakley*_*θ*=10_ with 77.5). This means that although these algorithms are highly effective in detecting epochs of sleep, they do not identify wake time so well, thus overestimating sleep epochs. This is a well-known behavior in the literature that is validated in our experiments,^11^ as seen by the low values of WASO (and the high values for sleep efficiency) when compared to the *Ground Truth*. *Scripps Clinic* algorithm achieved the highest *F*_1_ score, 81.8, which is not statistically different from the *Device Algorithm* (*p* = 0.47, *n* = 363), nor the *Manual Annotation* method (*p* = 0.10, *n* = 363).

On average, all results for the traditional algorithms are lower than both *Device Algorithm* and *Manual Annotation* baselines. This is somewhat expected as the *Device Algorithm* is optimized to be used with the particular actigraphy device employed in the experiments and the *Manual Annotation* resorts to human expert knowledge annotating the dataset.

The use of Webster’s rescoring rules shows gains in both specificity and precision for all the traditional algorithms but at the cost of sensitivity. This implies in a large proportion of epochs previously classified as *sleep* being reclassified as *awake*. For the top six traditional algorithms in terms of accuracy, *Resc. Oakley*_*θ*=40_, *Resc. Cole-Kripke*, *Resc. Scripps Clinic*, *Resc. Oakley*_*θ*=80_, *Resc. Sadeh*, and *Resc. Webster*, the use of rescoring rules resulted in higher accuracy and *F*_1_ scores. The opposite was found for the other two algorithms. The results show that the rescoring rules are, in general, effective in increasing the accuracy score (the average accuracy score increased from 75.1 to 78.0) but they should be applied with caution, as they could negatively impact the *F*_1_ score (average *F*_1_ score decreased from 80.3 to 79.8) or overestimate wake epochs (the group average WASO for the traditional algorithms went from 59 to 111 min). Note that there was no significant difference between WASO for *Resc. Scripps Clinic* and the *Ground Truth* (*p* = 0.901, *n* = 363). That was the case also for *Resc. Oakley*_*θ*=40_, (*p* = 0.07, *n* = 363), *Oakley*_*θ*=10_ (*p* = 0.13, *n* = 363), and *Perceptron* (*p* = 0.14, *n* = 363), for all the rest the differences were statistically significant.

Apart from the *Perceptron*, the ML algorithms have a very similar performance to each other for all the metrics evaluated. The sensitivity and *F*_1_ scores of the *Perceptron* algorithm were significantly lower than the second worst ML algorithm, *Linear SVM* (for both *p* < 0.001). *Perceptron* was also the only algorithm among the ML ones that overestimated WASO. The best ML algorithm with respect to accuracy score and *F*_1_ score, *Extra Trees*, was significantly better than the *Device Algorithm* (*p* < 0.001 for both accuracy and *F*_1_). While *Extra Trees* were significantly better than the *Manual Annotations* for accuracy (*p* = 0.016, *n* = 363), it was not significantly better for *F*_1_ (*p* = 0.26, *n* = 363).

Similar to the *Extra Trees* algorithm, the performance of DL algorithms were significantly better than the *Device Algorithm* for all metrics. Additionally, the *F*_1_ performance of LSTM 100, LSTM 50, CNN 100 and CNN 50 was also statistically better than *Manual Annotation* (*p* = 0.012, *p* = 0.046, *p* = 0.023, *p* = 0.039, ∀*n* = 363). Increasing the input size of both CNN and LSTM algorithms from 20 to 100 significantly increased the accuracy score (*p* = 0.014 for CNN and *p* = 0.035 for LSTM, ∀*n* = 363), but did not increase the *F*_1_ score significantly (*p* = 0.111 for CNN and *p* = 0.120 for LSTM, ∀*n* = 363). No significant differences were found between CNN 100 and LSTM 100 for accuracy and *F*_1_ (*p* = 0.789 and *p* = 0.817, ∀*n* = 363). All DL algorithms underestimated WASO and overestimated the sleep efficiency when compared to the *Ground Truth*.

The use of rescoring rules had a similar effect in both ML and DL algorithms as it did in the traditional algorithms: increased specificity and precision, but decreased sensitivity (i.e., increased WASO and decreased the sleep efficiency). This time, though, both accuracy and *F*_1_ went down after the usage of Webster’s rescoring rules, which indicate that these rules should not be used with ML and DL algorithms.

**Fig. 1:**
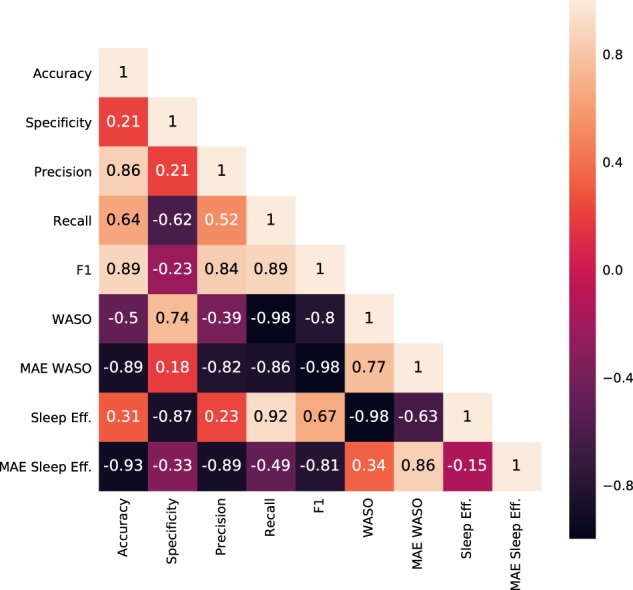
Pearson’s *r* correlation coefficients between the results of different metrics for Task Night (shown in Table 1) (*n* = 41)

In Fig. 1 we show the Pearson’s *r* correlation between the results of the 41 different algorithms shown in Table 1. The correlation coefficients show that *Sensitivity* is the metric that best (negatively) correlates with *WASO* and *sleep efficiency*, the sleep quality metrics studied in this work. However, an algorithm that reaches high values for WASO or sleep efficiency does not necessarily correctly assess one’s sleep quality. For that, we should rely on the mean absolute error between an algorithm and the ground truth for both WASO and sleep efficiency. The metrics that best correlated with MAE WASO is *F*_1_ (*r* = −0.98, *p* < 0.001), while the one that best correlates with MAE sleep efficiency is accuracy (*r* = −0.93, *p* < 0.001).

### Task Night&Day results: algorithm performance metrics during day time and night

Task Night&Day results are shown in Table 2. Once again the *Manual Annotation* method had the highest accuracy among the baseline methods. For Task Night&Day, though, the performance difference between the *Manual annotation* method and the *Device Algorithm* was high: for example, the *Manual Annotation* accuracy of 86.5 was 13% higher than *Device Algorithm*’s accuracy of 76.6. The accuracy results of *Always Wake* and *Always Sleep* show how the data now has more awake epochs (69.2%) than sleep ones.

**Table 2:** Results (Mean ± 95% confidence interval) for Task Night&Day

Method	Accuracy	Specificity	Precision	Sensitivity	F1
Baselines					
Manual annotations	**86.6** ± **2.2**	81.4 ± 2.9	**71.0** ± **4.0**	98.6 ± 1.0	**81.6** ± **3.0**
Device algorithm	76.6 ± 2.8	68.9 ± 3.7	58.6 ± 3.9	94.0 ± 1.7	71.2 ± 3.2
Always wake	69.2 ± 1.8	**100.0** ± **0.0**	0.0 ± 0.0	0.0 ± 0.0	0.0 ± 0.0
Always sleep	30.8 ± 1.8	0.0 ± 0.0	30.8 ± 1.8	**100.0** ± **0.0**	46.7 ± 2.2
Traditional algorithms					
Sazonov^9^	**82.7** ± **2.0**	**85.9** ± **2.5**	**70.9** ± **4.4**	75.5 ± 4.2	71.7 ± 3.9
Oakley * θ* = 10^32^	81.4 ± 2.3	79.1 ± 3.1	65.9 ± 4.0	86.8 ± 2.9	**73.7** ± **3.3**
Scripps Clinic^21^	77.4 ± 2.9	69.2 ± 3.9	59.5 ± 4.0	95.9 ± 1.5	72.4 ± 3.2
Oakley * θ* = 40^32^	76.2 ± 2.7	68.4 ± 3.7	58.1 ± 3.8	94.1 ± 1.6	70.9 ± 3.1
Cole-Kripke^6^	74.6 ± 2.9	64.9 ± 4.0	56.4 ± 3.8	96.6 ± 1.3	70.2 ± 3.2
Oakley * θ* = 80^32^	71.6 ± 2.9	60.5 ± 3.9	53.3 ± 3.6	96.7 ± 1.1	67.8 ± 3.1
Sadeh^5^	70.8 ± 3.2	58.7 ± 4.3	52.7 ± 3.7	**98.2** ± **1.1**	67.6 ± 3.3
Webster^28^	70.3 ± 3.2	58.0 ± 4.3	52.2 ± 3.6	98.1 ± 1.1	67.2 ± 3.2
Group average	75.6 ± 3.9	68.1 ± 8.4	58.6 ± 5.6	92.7 ± 6.6	70.2 ± 2.0
Rescoring rules applied to traditional algorithms					
Resc. Oakley *θ* = 10	**86.2** ± **1.9**	90.8 ± 2.3	79.5 ± 4.4	75.8 ± 4.0	76.0 ± 3.7
Resc. Scripps Clinic	85.8 ± 2.3	84.9 ± 3.1	73.7 ±4.5	87.6 ± 2.6	**78.8** ± **3.3**
Resc. Oakley * θ* = 40	85.3 ± 2.3	83.6 ± 3.1	72.0 ± 4.3	89.0± 2.2	78.6 ± 3.2
Resc. Cole-Kripke	84.8 ± 2.4	82.0 ± 3.3	70.8 ± 4.4	91.0 ± 2.1	78.6 ± 3.2
Resc. Oakley * θ* = 80	82.9 ± 2.6	78.3 ± 3.5	67.2 ± 4.3	93.2 ± 1.7	77.0 ± 3.2
Resc. Sadeh	82.6 ± 2.8	77.7 ± 3.8	67.1 ± 4.5	93.4 ± 1.9	76.9 ± 3.4
Resc. Sazonov	82.5 ± 1.9	**95.1** ± **1.5**	**80.2** ± **6.2**	53.5 ± 5.6	62.4 ± 5
Resc. Webster	82.2 ± 2.8	77.1 ± 3.8	66.4 ± 4.4	**93.8** ± **1.8**	76.5 ± 3.3
Group average	84.0 ± 1.4	83.7 ± 5.4	72.1 ± 4.5	84.7 ± 11.6	75.6 ± 4.5
Machine learning algorithms					
Extra trees	**86.7** ± **2.2**	**88.3** ± **2.6**	76.0 ± 4.8	82.3 ± 5.3	77.3 ± 4.8
Logistic regression	83.7 ± 2.6	79.1 ± 3.4	**67.9** ± **4.3**	94.3 ± 3.1	**77.6** ± **3.6**
Linear SVM	82.3 ± 2.7	76.7 ± 3.6	65.9 ± 4.2	**95.4** ± **2.7**	76.6 ± 3.5
Perceptron	79.7 ± 2.5	75.1 ± 3.3	62.7 ± 4.0	90.2 ± 2.6	72.9 ± 3.4
Group average	83.1 ± 4.6	79.8 ± 9.4	68.1 ± 9.0	90.5 ± 9.5	76.1 ± 3.5
Rescoring rules applied to machine learning algorithms					
Resc. logistic regression	**87.4** ± **2.2**	87.4 ± 2.6	76.3 ± 4.4	87.2 ± 4.4	79.7 ± 4.2
Resc. linear SVM	86.9 ± 2.2	86.1 ± 2.8	75.0 ± 4.5	**88.5** ± **3.8**	**79.7** ± **3.9**
Resc. perceptron	86.0 ± 2.1	87.4 ± 2.7	75.5 ± 4.5	82.8 ± 4.0	77.4 ± 3.8
Resc. extra trees	85.4 ± 2.0	**93.8** ± **1.9**	**80.9** ± **5.7**	64.3 ± 6.5	69.3 ± 6.1
Group average	86.4 ± 1.5	88.7 ± 5.54	76.9 ± 4.3	80.71 ± 17.81	76.5 ± 7.9
Deep-learning algorithms					
LSTM 100	**88.2** ± **2.0**	**88.9** ± **2.5**	**78.5** ± **4.3**	86.4 ± 3.9	80.8 ± 3.6
CNN 100	87.7 ± 2.3	86.6 ± 2.9	76.2 ± 4.4	**90.1** ± **4.1**	**80.8** ± **4.0**
CNN 50	87.6 ± 2.2	87.7 ± 2.7	76.7 ± 4.4	87.4 ± 4.2	80.1 ± 4.0
LSTM 50	86.4 ± 2.2	86.2 ± 2.9	74.9 ± 4.4	86.8 ± 3.2	79.1 ± 3.4
CNN 20	85.9 ± 2.2	86.1 ± 2.8	74.0 ± 4.4	85.5 ± 4.1	77.8 ± 3.9
LSTM 20	85.8 ± 2.2	87.2 ± 2.8	75.2 ± 4.5	82.5 ± 4.0	77.2 ± 3.8
Group average	86.9 ± 1.1	87.1 ± 1.1	75.9 ± 1.7	86.5 ± 2.6	79.3 ± 1.6
Rescoring rules applied to deep-learning algorithms					
Resc. CNN 100	**87.6** ± **2.0**	90.9 ± 2.2	80.4 ± 4.4	**80.0** ± **5.0**	**78.1** ± **4.5**
Resc. LSTM 100	87.5 ± 1.9	92.2 ± 2.0	**81.8** ± **4.3**	76.3 ± 5.0	77.0 ± 4.4
Resc. CNN 50	87.2 ± 1.9	92.1 ± 2.1	81.4 ± 4.3	75.9 ± 5.3	76.4 ± 4.8
Resc. LSTM 50	86.9 ± 1.9	91.5 ± 2.2	80.7 ± 4.4	75.8 ± 4.6	76.4 ± 4.0
Resc. CNN 20	86.0 ± 2.0	92.5 ± 2.0	81.1 ± 4.6	70.6 ± 5.4	73.3 ± 5.0
Resc. LSTM 20	84.7 ± 1.9	**93.6** ± **2.0**	82.1 ± 4.7	63.6 ± 5.5	69.3 ± 5.1
Group average	86.7 ± 1.2	92.1 ± 1.0	81.3 ± 0.7	73.7 ± 6.1	75.1 ± 3.4

Methods within each group are sorted by their mean accuracy score. Highest results for each category are marked in bold (*n* = 363)

Among the group of traditional algorithms, Table 2 shows that in terms of accuracy score, *Sadeh* and *Webster* fall short even to the *Always Wake* method by not being significantly different from it (*p* = 0.37 and *p* = 0.55, ∀*n* = 363). *Sazonov* achieved the highest accuracy score for Task Night&Day (82.7), even though it did not do well for Task Night, having an accuracy score lower than the *Device Algorithm* for Task Night. *Oakley*_*θ*=10_ and *Scripps Clinic* algorithms were the only ones that outperformed the *Device Algorithm* baseline for accuracy in both tasks.

The rescoring rules applied in addition to the traditional algorithms improved both *accuracy* and *F*_1_ score for all methods in Task Night&Day, contrary to the results of Task Night, with the sole exception of accuracy and *F*_1_ score for *Resc. Sazonov*, which decreased, respectively, from 82.7 to 82.5 and from 71.7 to 62.4. The minimum accuracy of the rescoring methods was found for *Resc. Webster* (82.2), which was still significantly higher than the accuracy of the *Device Algorithm* (76.5, *p* = 0.005, *n* = 363).

The performance of the ML algorithms shown in Table 2 for Task Night&Day is similar to the performance of the same algorithms for Task Night, i.e., the ranking with regards to accuracy and *F*_1_ for the four ML algorithms studied was the same: *Extra Trees* followed by the *Logistic Regression*, *Linear SVM* and the *Perceptron*. The best accuracy of 86.7 for the *Extra Trees* was significantly higher than the *Device Algorithm* (*p* < 0.001, *n* = 363), but not significantly different from the *Manual Annotation* (*p* = 0.95, *n* = 363).

The rescoring methods applied in addition to the ML algorithms improved the accuracy and *F*_1_ scores for all but one method, the *Extra Trees*. The highest accuracy was 87.3 for the *Resc. Logistic Regression*, which was significantly higher than the *Manual Annotation* (*p* < 0.001, *n* = 363), while its *F*_1_ was not found significantly different from the *Manual Annotation* (*p* = 0.79, *n* = 363). Table 2 also shows that the average accuracy of the ML group went from 83.1 to 86.4 (improvement of 3%) when using the rescoring rules.

Table 2 shows that the DL algorithms can reach an accuracy as high as 88.2 for *LSTM 100* without rescoring rules and 87.6 for *CNN 100* with rescoring rules. *LSTM 100*, *CNN 100*, and *CNN 50* were the only approaches performing better than the *Manual Annotation* for accuracy, but the difference was not statistically significant (*p* = 0.287, *p* = 0.495, and *p* = 0.528, ∀*n* = 363). Finally, similar to Task Night, differences between CNN 100 and LSTM 100 were not significant for accuracy (*p* = 0.734, *n* = 363) and *F*_1_ (*p* = 0.995, *n* = 363).

## Discussion

In this study, we introduced a new benchmark for sleep-wake scoring algorithms, based on data from the MESA Sleep study.^17,18^ While the original MESA Sleep dataset can be obtained upon request from https://sleepdata.org/datasets/mesa, we make freely available for download all the scripts required to process the data and generate the same datasets and results reported here for both Task Night and Night&Day, at https://github.com/qcri/sleep_awake_benchmark. By providing this resource, we hope that future research in developing new sleep-wake scoring can be easily facilitated.

The results of our experiments showed that the proprietary algorithm used by the actigraphy device, although likely optimized for it, did not perform the best for Task Night and Night&Day. The average accuracy and *F*_1_ scores achieved by both *Oakley*_*θ*=10_ or *Scripps Clinic* algorithms were higher than the *Device Algorithm*.

Our experiments validated the use of four ML algorithms, which presented statistically significant improvements compared to the *Device Algorithm*. It must be noted that in this work we devised only features based on the distribution of activity counts. In the Supplementary Material, we show our initial experiments with features extracted from the demographic and clinical information of the participants. More complex feature engineering, which can likely improve the current results even further, is left as future work.

Furthermore, we evaluated two state-of-the-art deep- learning techniques (DL), such as CNN and LSTM. Owing to the success of DL algorithms in areas such as computer vision, speech recognition, and bioinformatics, new architectures are continually being proposed. The use of a benchmark like the one proposed in this paper can potentially accelerate the adoption of new techniques in the sleep science field. Most of the traditional algorithms, as well as the features devised in this work for the traditional ML and DL algorithms, make use of future activity counts, i.e., when predicting the epoch *n*, these algorithms use the activity counts in proceeding epochs, *n* + 1, *n* + 2, and so on. The only exception is the *Sazonov* algorithm (refer to the Supplementary Material for the description and formula of each algorithm). Real-time applications should not use future activity counts. However, the typical usage of sleep-wake scoring algorithms does not require real-time predictions.

The experiments with sleep quality metrics in Task Night show that the choice of scoring algorithm can highly influence the interpretation of people’s sleep behavior. For example, while the mean sleep efficiency of our cohort is 58% (as shown by the *ground-truth*), the device algorithm reports a sleep efficiency of 73%. An algorithm that systematically overestimates sleep efficiency might fail to identify and report sleep-related diseases. Conversely, an algorithm that systematically underestimates sleep efficiency might cause too many false-positive, which can lead to unnecessary clinical evaluations. Noteworthy, the traditional formulas showed a larger variance in terms of WASO (from 26 min to 149 min) and sleep efficiency (from 54 to 82%) than ML and DL algorithms. For sleep efficiency, ML algorithms vary only from 61 to 66%, while the DL algorithms vary from 64 to 67%. Finally, significant improvements in the clinical metrics can be achieved by new algorithms. When using the device algorithm, the average absolute error of sleep efficiency compared to the ground truth is 17pp, while the average error for WASO is 53 min. By using the best DL algorithm, LSTM 100, the sleep efficiency error goes down to <10pp (70% better) while WASO error goes down to 44 min (20% better).

The Task Night, which studies sleep-wake scoring algorithms to be exclusively used during sleep, and Task Night&Day, which studies sleep-wake algorithms to be used on a 24-h period, are not the only possibilities with this dataset. Other tasks, such as predicting the sleep and awake onset, which are essential for the assessment of sleep quality, are left as future work as there is a wide range of potential sleep quality metrics. Nevertheless, the results of Task Night and Night&Dday show that those tasks have significant differences. For example, our experiments show that the use of Webster’s rescoring rules should be limited to the traditional algorithms for Task Night, while they worked well for most of the algorithms for Task Night&Day, avoiding overestimation of sleep. Based on our results, we advocate that the algorithm of modern actigraphy devices and wearables could adaptively switch from an algorithm specialized for the night (as in Task Night) to another specialized for the day (as in Task Night&Day) depending on the time of the day.

A primary limitation of our work is that, although the MESA Sleep study includes a diverse population from different ethnicities, it is exclusively composed of adults. An ideal cohort for sleep-wake scoring should include other populations, such as toddlers, kids or adolescents, as well as, people with disorders that affect movements, such as Parkinson’s disease,^24^ or specific sleep disorders, such as insomnia,^25^ sleep apnea or restless legs syndrome. The expansion of the cohort proposed in our work is highly desirable and appreciated, but is left as future work. Additionally, actigraphy as a device is incapable of discriminating the different sleep stages (e.g., sleep stage 1, stage 2, stage 3, and REM sleep). Another minor limitation is that the nights for which the participants undergo PSG are usually easier to interpret than nights “in the wild” as PSG imposes a normal sleep and wake time, which may be absent for some of these subjects. An additional constraint of this study is that it is based only on one actigraphy device (Philips Actiwatch Spectrum). We need to be aware that the device used can impact the generalization of the results. Although some studies use consumer-grade devices, there are concerns with the accuracy of these devices, among other factors.^26^

Finally, given the great importance of sleep to health and human functioning, developing accurate analytic approaches for actigraphy data is the key to precisely determine sleep quality. This is also important given the increasing use of wearable devices that use different algorithms to assess and optimize sleep.

## Methods

The methodology used in this study has several components, which are introduced in this section. The main component of this study is the MESA Sleep dataset. In a dataset such as this one, researchers usually devise and evaluate new algorithms to score nocturnal sleep-wake epochs. For our study, we identified this common task as Task *Night* and proposed to extend the benchmark of such algorithms to a 24-h period, our Task *Night&Day*. The use of large datasets opens the possibility of validating, comparing and evaluating new approaches, in particular, machine learning algorithms. This section also describes the state-of-the-art approaches validated in this work and evaluation metrics used in our experiments.

### MESA sleep dataset

The MESA Sleep^17,18^ experiments were conducted using the Compumedics Somte System to record PSG data and the Actiwatch Spectrum, Philips Respironics to record actigraphy data. The data was acquired in six field centers that are located at different places across the United States.Institutional review board approval was obtained at each participating center and written informed consent was obtained from all participants.^17,18,27^

In this work, we used the synchronized PSG and actigraphy data for 1817 subjects out of the initial 2237 subjects that participated in the MESA Sleep study. The data from the other 420 subjects were discarded because of at least one of the following reasons: (1) PSG and actigraphy studies did not occur concurrently;^20^ (2) data failed the minimal actigraphy or PSG quality standard (i.e., <3 h of useable data);^20^ (3) PSG recorded for over 16 h. This last criterion was adopted in this work to increase the quality of the dataset by making it consistent w.r.t. all participants. This resulted in the removal of only nine participants.

The PSG and actigraphy records were synchronized in 30-s epochs, and the 1817 subjects were randomly split into a training set of 1454 subjects—80% of the subjects—and a test set of 363 subjects—the other 20%. The training set was used to tune and optimize model hyperparameters, i.e., the set of tunable parameters that control the quality of the model (e.g., number of leaves in a tree, the learning rate of an algorithm) in terms of training accuracy, generalization performance and prevention of over/under-fitting, while the test set was used to obtain the results reported in this work. A summary of the statistics for the training and test set are shown in Table 3.

**Table 3:** Summary statistics of the MESA Sleep dataset

Dataset	Total	Female	Male	White	Chinese	Black	Hispanic	Age (mean ± Std.)	Min. age	Max age
Training	1454	799 (55%)	539 (37%)	157 (11%)	404 (28%)	354 (24%)	655 (45%)	69.36 ± 9.18	55	94
Test	363	186 (51%)	177 (49%)	126 (35%)	44 (12%)	102 (28%)	91 (25%)	69.24 ± 8.79	54	92

### Sleep-wake tasks

In this work, we propose two complementary tasks using the synchronized PSG and actigraphy data of the MESA Sleep dataset.

#### Task Night

Traditionally, actigraphic sleep-wake scoring algorithms are compared to PSG gold standard with overnight experiments only (e.g., refs ^5,6,28^). For instance, apart from the Granovsky algorithm^13^ of Table S1, all others were devised and optimized for scoring sleep-wake patterns during the period that PSG is also used. We name this typical task—the direct comparison of actigraphy algorithms with PSG—as Task Night. Note that this is also the usual setting in validation studies (e.g., refs ^7,8,22^). As common in the literature, the activity counts are adjusted to 30-s epochs and synchronized to the PSG signals. The PSG-identified sleep periods (sleep phases 1, 2, 3, 4, and rapid eye movement (REM)) were scored as *sleep*, while awake periods were scored as *wake*. These two non-overlapping periods are coded here into numerical scores: 1 for *sleep* and 0 for *wake*. With recordings starting when the PSG equipment was turned on, and finishing when the PSG was turned off, a total of 2,266,659 30-s epochs were recorded from the 1817 subjects. Thus, our training dataset (80% of the whole cohort) comprised of over 1.75 million samples.

#### Task Night&Day

During night time, traditional actigraphy scoring algorithms are known for their high sensitivity (i.e., algorithms score most of the actual sleep as sleep) and low specificity (i.e., a limited proportion of all epochs are classified as wake^8,29^). During the day, actigraphy scoring algorithms over detect naps, as epochs with low activity tend to be scored as sleep.^30,31^ In Task Night&Day, we propose to investigate the behavior of different scoring algorithms both during night and day by extending the Task Night data to include epochs before and after the use of PSG. We include all actigraphy data recorded up to 8 h before and after PSG was conducted. While PSG annotations are the gold standard used in Task Night, these annotations are not provided to individuals during the day. Instead of simply assuming that individuals are awake in the 8 h after PSG was conducted, we take advantage of the manual annotations that are provided in the original MESA dataset. The expert annotations were collected following a clinical research protocol in which the expert is instructed to set the beginning and end of the rest interval based on multiple signals, which include drops/increases in activity counts, as well as event markers, sleep diaries, and light levels.^19,20^ Two experts scored the MESA dataset with an inter-scorer reliability larger than 90% (*n* = 19).^19,20^ We assume that all epochs during naps in the day period were sleep epochs.

**Fig. 2:**
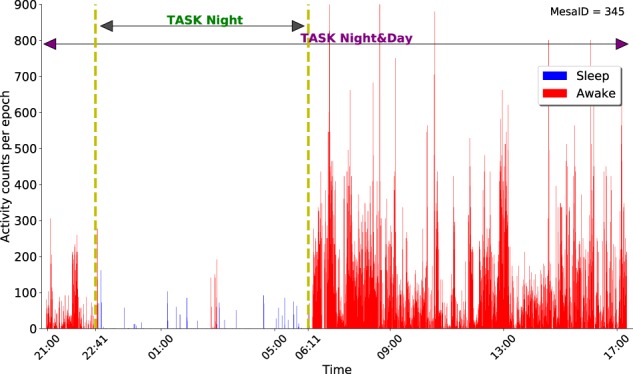
Activity counts by time for MesaID 345. Each point corresponds to the activity count measured by the actigraphy device for an interval of 30 s. The yellow lines mark the borders of the data used for Task Night—the start and end of PSG period (in this case, from 9:15 p.m. to 09:24 a.m.). The extended period before and after the use of PSG (from 9:00 p.m. to 6:00 p.m. in the next day) is the data used for Task Night&Day

Figure 2 shows the activity counts for one subject (MesaID 345) randomly selected from the dataset. The data lying between the yellow lines correspond to Task Night: it is the time range when both actigraphy and PSG were used. The extended data outside this range (up to 8 h before and after PSG) is also used in Task Night&Day. Note that in this example, the subject started using the actigraphy device just a few hours before the sleep period was recorded with the PSG.

### Scoring algorithms

In this section, we start by discussing the role of traditional sleep-wake scoring algorithms. We also present the rescoring rules, a set of rules to amend known shortcomings of the traditional algorithms, as well as machine learning approaches. All the reviewed algorithms are systematically evaluated in our experiments.

#### Traditional algorithms

A number of sleep-wake scoring algorithms were devised in the previous 40 years. These scoring algorithms aim to estimate whether the user wearing the actigraphy device was asleep or awake at a given epoch *T* based on the activity counts measured by the actigraphy device.

In this work, we study six of these algorithms: *Webster*,^28^ *Cole-Kripke algorithm*,^6^ *Sadeh*,^5^ *Oakley*,^32^ *Sazonov*,^9^ *Scripps Clinic*.^21^ The historical information about each one of these algorithms, as well as their details, is described in the Supplementary Material. Also note that Table S1 summarizes and compares the datasets used to devise the traditional algorithms to the dataset used in this work.

#### Rescoring rules

Webster et al.^28^ detected that the most common error in their scoring method was scoring wake as sleep. They proposed a set of simple rescoring rules to correct for such systematic errors. Their set of rules were posteriorly validated by different researchers.^33^

In this work, we systematically evaluate their set of rules by applying them to each of the evaluated scoring methods. Their five rules can be defined as: (R1) after at least four epochs scored as wake, the first epoch scored sleep is rescored wake; (R2) after at least ten epochs scored as wake, the first three epochs scored sleep are rescored wake; (R3) after at least 15 epochs scored as wake, the first four epochs scored sleep are rescored wake; (R4) six epochs or less scored sleep surrounded by at least ten epochs (before or after) scored as wake are rescored wake; and (R5) ten epochs or less scored as sleep surrounded by at least 20 epochs (before or after) scored as wake are rescored wake. These five rules were applied sequentially from (R1) through (R5) as previously done by Cole et al.^6^.

#### Machine learning algorithms

Machine learning (ML) and deep-learning (DL) techniques have been successively used in many domains, including sleep science,^11,13^ to discover and classify patterns in the data. These techniques aim to *learn* with data, i.e., they create a mathematical model after a number of learning examples (training set). These learned models can be used to make predictions when a new set of data is used (test set). Tilmanne et al.,^11^ for example, investigated the use of two ML techniques, Multilayer Perceptrons and Decision Trees, as sleep-wake scoring algorithms, finding them more accurate than Sazonov and Sadeh’s algorithms. Granovsky et al.^13^ employed a state-of-the-art DL technique, Convolutional Neural Networks (CNN),^34^ to score sleep-wake stages based on actigraphy data of 35 chronic cluster headache patients. Granovsky et al. results are promising, although, different from all other related work, their evaluation was not conducted with PSG as ground truth, thus not as fine-grained.

In this work, we evaluate both ML and DL techniques. We investigate a variety of ML techniques: Logistic Regression,^35^ Support Vector Machines (SVM),^36^ Extra Trees^37^, and Perceptron,^38^ all of which have been successfully employed in tasks in the bioinformatics domain, such as protein function prediction,^39,40^ gene regulatory network inference^41–43^ and human activity prediction.^44^ In case of DL techniques, we investigate Convolutional Neural Networks (CNN), which can capture local contextual features, and long short-term memory (LSTM)^45^ recurrent network, which can not only capture local information but also retain long-term dependencies.

The feature set used by the ML techniques follows previous work.^11^ We manually devised a total of 370 features based on the raw signal extracted from the actigraphy device. For the current epoch *T*, apart from the raw value and natural logarithm value of the activity count at *T*, features are based both on a centered and non-centered (i.e., considering only activity counts in previous epochs) sliding window of N epochs (with 1 ≤ *N* < 20). For each sliding window, we calculated summary statistics such as the mean, variance and standard deviation of the activity counts of the window.

Owing to the fact that the DL techniques used—CNN and LSTM—are able to infer new features from the data, their input is a window of a fixed size (either 20, 50 or 100) containing the raw signal from the actigraphy device. When we run multi-layered CNNs with multiple filters, we capture non-linear interactions between adjacent raw activity counts and obtain a new vector space representation for the raw signals. Similarly, with LSTMs, we abstract long-term and short-term raw activity-based non-linear dependencies in a new vector space, which helps to discriminate sleep stage from wake state.

A full list and examples of the features set used in this work is presented in the Supplementary Material.

### Evaluation metrics

In this work, we adopted commonly used metrics to evaluate the performance of the scoring algorithms: accuracy, sensitivity, specificity, precision, *F*_1_ score, area under the receiver operating curve, and area under the precision-sensitivity curves. As done in other works in the literature,^8,11,12,22^ the sleep-wake scoring task is treated as a binary classification in which the positive label is sleep and the negative label is awake. This way, an algorithm with a high score for precision, for example, is an algorithm that correctly classifies sleep epochs as sleep. A detailed description of the metrics is shown in the Supplementary Material. Tests of statistical significance were conducted with a two-tailed *t*-test.^46^

In particular, for Task Night, we investigated two additional metrics for *sleep quality*, which are of clinical relevance. They are the number of *minutes* wake after sleep onset (WASO) and the sleep efficiency. Sleep efficiency is calculated as the *percentage* of sleep epochs in the entire record. We used the first epoch recorded as sleep by PSG as sleep onset epoch for WASO, whereas we used the entire record to calculate sleep efficiency. Both metrics are frequently used in the literature.^6,22,47,48^

We also calculated the mean absolute error (MAE) between WASO and sleep efficiency across participants for each algorithm, comparing the performance of an algorithm with the ground truth data.

### Baseline

In our experiments for both Task Night and Night&Day, we use four different baselines for comparison: (1) *Device algorithm*: the proprietary algorithm of the actigraphy device used in the MESA Sleep experiments—MESA documentation states that Oakley *θ* = 40 was the algorithm used by the device;^20^ (2) *Manual Annotation*: the manual annotation made by an expert without knowledge of the PSG annotations, solely based on the device algorithm, participant’s sleep journals and variables such as the activity patterns and the time of the day^19,20^ – the same used in Task Night&Day for the day period; (3) *Always Sleep*: an algorithm that classifies any epoch as Sleep; and (4) *Always Wake*: an algorithm that classifies any epoch as Wake. Additionally, for Task Night, we show the performance of an oracle method that always predicts the correct labels (*Ground Truth*). This is useful to inspect the expected values for WASO and sleep efficiency metrics.

### Reporting summary

Further information on research design is available in the Nature Research Reporting Summary linked to this article.

## Supplementary information

Supplementary Material

reporting summary

## Data Availability

The original MESA Sleep dataset can be obtained upon request from https://sleepdata.org/datasets/mesa.

## Supplementary information

**Supplementary information** accompanies the paper on the *npj Digital Medicine* website (10.1038/s41746-019-0126-9).

## Code availability

We make freely available for download all the scripts required to process the data and generate the same datasets and results reported here for both Task Night and Night&Day at https://github.com/qcri/sleep_awake_benchmark.
