# Supplementary material for: Benchmark on a large cohort for sleep-wake classification with machine learning techniques
Source: NPJ Digit Med. 2019 Jun 7;2:50. doi: 10.1038/s41746-019-0126-9 (PMC6555808; doi:10.1038/s41746-019-0126-9)
Supplement: Supplementary file 1 — Supplementary Material [file 41746_2019_126_MOESM1_ESM.pdf]

Supplementary Material: Benchmark on a Large Cohort for Sleep-Wake classification with Machine Learning Techniques

Joao Palotti *† jpalotti@hbku.edu.qa
Raghvendra Mall† rmall@hbku.edu.qa
Michael Aupetit† maupetit@hbku.edu.qa
Michael Rueschman‡§ mrueschman@bwh.harvard.edu
Meghna Singh ¶ singh742@umn.edu
Aarti Sathyanarayana§ aarti.sathyanarayana@childrens.harvard.edu
Shahrad Taheri‖ szt2004@qatar-med.cornell.edu
Luis Fernandez-Luque*† lluque@hbku.edu.qa

*Corresponding Author
†Qatar Computing Research Institute, HBKU, Qatar
‡Brigham and Women's Hospital, Boston, USA
§Harvard University, Boston, USA
¶University of Minnesota, Minneapolis, USA
‖Weill Cornell Medicine Qatar, Qatar

Supplementary Methods

Traditional Sleep-Awake Algorithms

In this work, we study six traditional sleep-wake scoring algorithms frequently used in the literature and by commercial actigraphy devices: *Webster*,[1] *Cole-Kripke algorithm*,[2] *Sadeh*,[3] *Oakley*,[4] *Sazonov*,[5] *Scripps Clinic*.[6]

Supplementary Figure 1 shows typical components seen in most of the traditional algorithms. For example, the raw activity counts extracted from the sensor for the epoch T being assessed are represented by A_T. Similarly, A_{T+1} and A_{T-1} are, respectively, the activity counts for epoch succeeding and preceding epoch T. The length of an epoch in all experiments conducted in this work is 30-seconds, the usual epoch length of PSG devices.

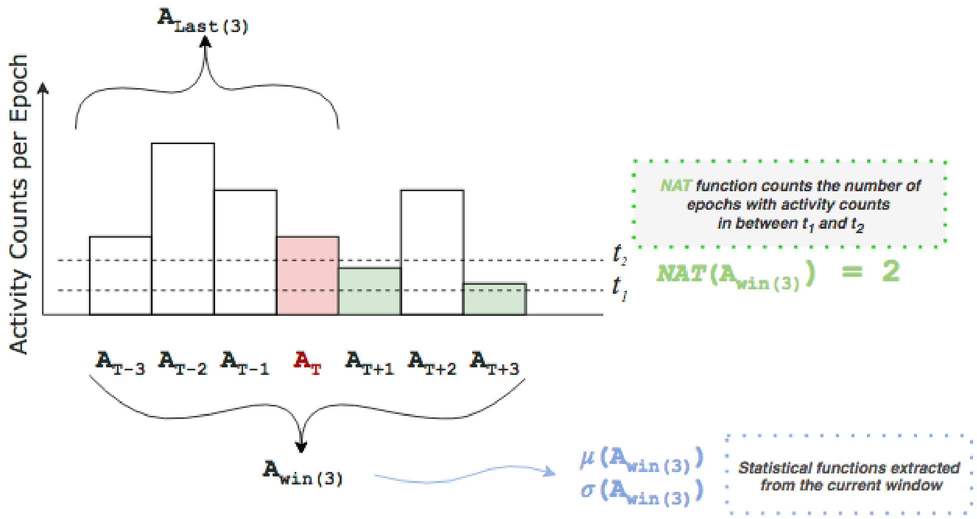

Supplementary Figure 1: Typical components of scoring algorithms are shown in this figure. A_T is the raw activity counts of epoch T, which has to be scored as *sleep* or *wake* by a scoring algorithm.

Some algorithms rely on mathematical functions applied to the distribution of activity counts. To create such a distribution, we use functions such as *Win(δ)* and *Last(δ)*. *Win(δ)* represents a time window of all activity counts between T-δ and T+δ, inclusively, for the current epoch T. Likewise, *Last(δ)* comprises of the distribution of activity counts from T-δ to T, inclusively. In Supplementary Figure 1, we highlight the activity counts that would be used in each distribution when $\delta = 3$. As mathematical functions, not only well-known functions such as the mean (μ) and the standard deviation (σ) were used, but also a new function named *NAT*, which counts, in a given distribution, the number of epochs with equal activity counts between two thresholds t_1 and t_2.

In the pioneering work of Webster et al.,[1] an early version of actigraphic devices showed the feasibility of using an activity-based sleep monitor system to quantify sleep time with reasonable accuracy, while avoiding the impracticality and expense of PSG. Exclusively based on the raw activity counts of surrounding epochs of epoch T, Webster et al. algorithm can be defined as the following formula:

$$W(T) = 0.025 \times (0.15 \times A_{T-4} + 0.15 \times A_{T-3} + \\ 0.15 \times A_{T-2} + 0.08 \times A_{T-1} + 0.21 \times A_T + \\ 0.12 \times A_{T+1} + 0.13 \times A_{T+2}) \quad (1)$$

An epoch T is scored as sleep if $W(T) < 1$ and wake if $W(T) \geq 1$. We refer to this formula in this work as *Webster*.

Validating and expanding the experiments of Webster, Cole et al.[2] proposed the following linear regression

model based on the same parameters explored by Webster et al.:

$$C(T) = 0.00001 \times (404 \times A_{T-4} + 598 \times A_{T-3} + 326 \times A_{T-2} + 441 \times A_{T-1} + 1408 \times A_{T} + 508 \times A_{T+1} + 350 \times A_{T+2}) \quad (2)$$

Similar to *Webster*, an epoch T is scored as sleep if $C(T) < 1$ and wake, otherwise. This algorithm is commonly referred as *Cole-Kripke algorithm* in the literature and we keep the same terminology in this paper.

Based on quantities that can be derived from a distribution of activity counts, such as the *mean*(μ) or *standard deviation* (σ), Sadeh et al.[3] introduced a new formula for sleep-wake scoring in 1994. Given ln as the natural logarithm function, their formula can be defined as:

$$S(T) = 7.601 - 0.065 \times \mu(A_{Win(5)}) - 1.08 \times NAT(A_{Win(11)}) - 0.056 \times \sigma(A_{Last(6)}) - 0.703 \times \ln(A_T + 1) \quad (3)$$

An epoch T is scored as sleep if $S(T) > 0$ and wake, otherwise. We refer to this formula as *Sadeh*.

Similar to Webster's and Cole's, Oakley et al.[4] introduced a formula based on the activity counts of the surrounding four epochs:

$$O(T) = 0.04 \times A_{T-4} + 0.04 \times A_{T-3} + 0.20 \times A_{T-2} + 0.20 \times A_{T-1} + 2.00 \times A_{T} + 0.20 \times A_{T+1} + 0.20 \times A_{T+2} + 0.04 \times A_{T+3} + 0.04 \times A_{T+4} \quad (4)$$

Oakley et al. classifies epochs that fall under a specified sleep/wake threshold value Th as sleep. Three threshold values were experimented by them: $Th_{low} = 20$, $Th_{mid} = 40$, and $Th_{high} = 80$. In this paper, we exhaustively experimented all possible integers Th, for $Th \in \{0, 5, 10, 15, ..., 300\}$. An epoch T is scored as sleep if $O(T) \leq Th$ and wake, otherwise. We refer to this formula in this paper as *Oakley*.

Sazonov et al.[5] was the first study to investigate the use of Machine Learning techniques for the sleep-wake scoring problem. To score an epoch T, they trained a Logistic Regressor based on the activity counts of epoch T and the previous δ epochs. In their work, the following formula was devised:

$$Z(T) = 1.727 - 0.256 \times A_T - 0.154 \times A_{T-1} - 0.136 \times A_{T-2} - 0.140 \times A_{T-3} - 0.176 \times A_{T-4} \quad (5)$$

An epoch T is scored as sleep if $Z(T) > 0.5$ and wake, otherwise. We refer to this formula in this paper as *Sazonov*.

More recently, Kripke et al.,[6] based on the sum of activity counts for a window of 21 epochs, devised the following formula (the weights of some epochs were set to 0.0):

$$K(T) = 0.204 \times (0.0064 \times A_{T-10} + 0.0074 \times A_{T-9} + 0.0112 \times A_{T-8} + 0.0112 \times A_{T-7} + 0.0118 \times A_{T-6} + 0.0118 \times A_{T-5} + 0.0128 \times A_{T-4} + 0.0188 \times A_{T-3} + 0.0280 \times A_{T-2} + 0.0664 \times A_{T-1} + 0.0300 \times A_{T} + 0.0112 \times A_{T+1} + 0.0100 \times A_{T+2}) \quad (6)$$

An epoch T is scored as sleep if $K(T) < 1.0$ and wake, otherwise. This formula is commonly referred as *Scripps Clinic* and we keep the same terminology in this paper.

Supplementary Table 1 summarizes and compares the datasets used to devise these algorithms to the MESA dataset used in this work.

Supplementary Table 1: Summary of related work and size comparison between the dataset used in this work and used in other work. Studies were divided between the original papers in which scoring methods were devised and further papers that validated those scoring algorithms.

Reference	Participants		#Epochs	Device(s) used in the experiment (Manufacturer)	Observations
	Training	Test			
**This Work**					
MESA Task Night	1,454	363	2,266,659	Actiwatch Spectrum (Respironics)	Actigraphy period aligns with PSG
MESA Task Night&Day	1,454	363	4,453,428		MESA Task 1 plus wake hours before/after the use of PSG
**Studies Proposing New Sleep-Wake Scoring Algorithms**					
Webster et al.[1]	17	3	13,488	N.A.	Devised the Webster Algorithm and re-scoring rules
Cole et al.[2]	20	21	18,770	Motionlogger Actigraph (Ambulatory Monitoring)	Devised the Cole Algorithm
Sadeh et al.[3]	36	10	5,066	N.A.	Devised the Sadeh Algorithm
Oakley et al.[4]	N.A.	N.A.	N.A.	Sleepwatch (Ambulatory Montoring), Actiwatch and Actical (Respironics)	Formula used from Tonetti et al.[7]
Sazonov et al.[5]	4	4	N.A.	N.A.	Devised the Sazonov Algorithm
Kripke et al.[6]	49	49	N.A.	Actiwatch-L and Actiwatch Spectrum (Respironics)	Devised the Kripke Algorithm
Tilmannee et al.[8]	248	106	336,958	N.A.	Compared the use of Saznov and Sadeh to ML algorithms
Granovsky et al.[9]	20	5	N.A.	Actiwatch - No details (Respironics)	Deep Learning algorithms to score sleep-wake epochs; Not validated against PSG.
**Studies Validating/Comparing Existing Sleep-Wake Scoring Algorithms**					
Kushida et al.[10]	-	100	N.A.	Actiwatch 4 (Respironics)	Validated Oakley algorithm with PSG
Jean-Louis et al.[11]	-	5	N.A.	Actillume and Mini Motionlogger (Ambulatory Monitoing)	Compared PSG to Cole algorithm
De Souza et al.[12]	-	21	N.A.	Mini Motionlogger (Ambulatory Monitoring)	Compared PSG to Cole and Sadeh algorithms
Tonetti et al.[7]	-	12	N.A.	Mini Motionlogger (Ambulatory Monitoring) and Actiwatch (Respironics)	Compared Sadeh and Oakley to PSG
Weiss et al.[13]	-	30	N.A.	Sleepwatch (Ambulatory Monitoring), Actiwatch and Actical (Respironics)	Compared PSG to thresholds of the Oakley algorithm
Tudor-Locke et al.[14]	-	30	N.A.	GT3X+ (ActiGraph LLC)	Compared Sadeh to manually labeled actigraphic data
Marino et al.[15]	-	77	232,849	Actiwatch 64 and the Actiwatch Spectrum (Respironics)	Compared PSG to Cole algorithm
Patel et al.[16]	-	50	N.A.	Actiwatch Spectrum (Respironics)	Compared Oakley to manually labeled actigraphic data
Quante et al.[17]	-	22	N.A.	GT3X+ (ActiGraph LLC) and Actiwatch Spectrum (Respironics)	Compared Sadeh and Cole to PSG

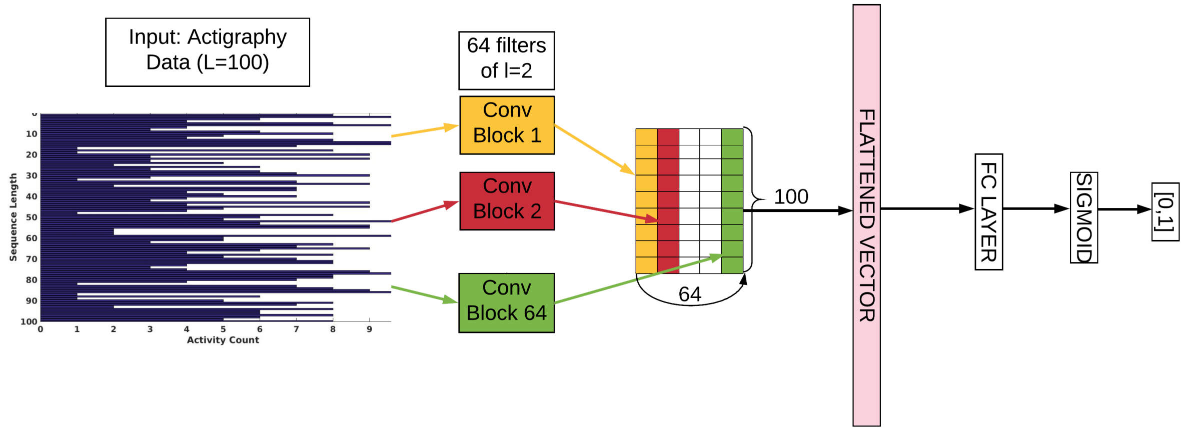

Supplementary Figure 2: The raw activity counts captured by the actigraphy device are used as input to the convolution neural network. We set the parameters of this network to have sixty-four convolution blocks which are flattened and transformed into binary predictions with a Sigmoid function.

Machine Learning Algorithms

Feature set for Standard Machine Learning Algorithms

A feature set is a representation of the object that we are classifying. A necessary first step for any ML and DL algorithm is the modeling of features for the given task. In this work, we model different features for the ML and DL algorithms as described below.

In the context of sleep-wake scoring, features for the ML algorithms are inspired by the traditional sleep-wake scoring formulas, previously seen in Section . For the current epoch T, apart from the raw value and natural logarithm value of the activity at T, features are based both on a centered and non-centered (i.e., considering only activity counts in previous epochs) sliding window of N epochs (with $1 \leq N < 20$), $A_{Win(N)}$ and $A_{Last(N)}$ respectively (e.g., used in Sadeh's algorithm, Equation 3). For each sliding window type (centered and non-centered) and value of N, we calculated the following statistics: mean, median, standard deviation, max, min, variance, NAT, and $AnyAct$. NAT,[3] as in Equation 3, counts the number of epochs in the sliding window with the activity counts between 50 and 100, inclusively. $AnyAct$ counts the number of epochs in the sliding window with activity counts greater than 0. Additionally, we calculate the *Skewness* and *Kurtosis* of the values in a sliding window. These can provide useful insights about the distribution of values in that window (e.g., a positive skew, characterized by the fact that the right tail of the distribution is longer, might indicate that the user of the actigraphy device is stopping a physical activity or lying in bed to rest). Supplementary Figure 3 exemplifies how the features used by the ML algorithms were extracted from the raw signal captured by the actigraphy device. A complete list of all 370 features used for the ML algorithms in this work is shown in Supplementary Table 2.

The DL techniques that we validate in this work allow us to have, as input, the time series of raw activity counts. This is possible because DL techniques are able to infer new features from the raw data. Therefore, instead of using a transformation of the data (as done for the ML algorithms), we use centered windows with the raw activity counts of the past and future X epochs. We tested three different values for X: 20, 50 and 100. In this work, we employed commonly used settings for Convolutional Neural Networks (CNN) and Long Short-Term Memory (LSTM) recurrent network. The multi-layered CNN used in this work can capture non-linear interactions between adjacent raw activity counts and obtain a new latent space representation for the raw signals. Similarly, the LSTMs abstract long-term and short-term raw activity based non-linear dependencies in a new latent space which helps to discriminate sleep stage from wake state. Supplementary Figure 2 shows the setting of the CNN architecture with X=100, LSTM settings are similar.

Supplementary Table 2: Details on the 370 features devised to represent each activity count.

Metric	Type	Description	Number of Features		
			Past Windows	Centered Window	Total
Activity Count	Int	Raw activity count from the actigraphy device		1	
Logarith Activity Count	Float	Natural logarithm of the activity count		1	
Mean	Float	Mean value for the window of activity of size X. $1 \leq X < 20$	19	19	38
Median	Float	Median value for the window of activity of size X. $1 \leq X < 20$	19	19	38
Std	Float	Standard deviation value for the window of activity of size X. $1 \leq X < 20$	19	19	38
Var	Float	Variance value for the window of activity of size X. $1 \leq X < 20$	19	19	38
Min	Int	Minimum value for the window of activity of size X. $1 \leq X < 20$	19	19	38
Max	Int	Maximum value for the window of activity of size X. $1 \leq X < 20$	19	19	38
NAT	Int	NAT value for the window of activity of size X. $1 \leq X < 20$	19	19	38
Any Activity	Int	Number of epochs with any activity in the window of size X. $1 \leq X < 20$	19	19	38
Skewness	Float	Skewness $4 \leq X < 20$	16	16	32
Kurtosis	Float	Kurtosis $4 \leq X < 20$	16	16	32
		Total		370	

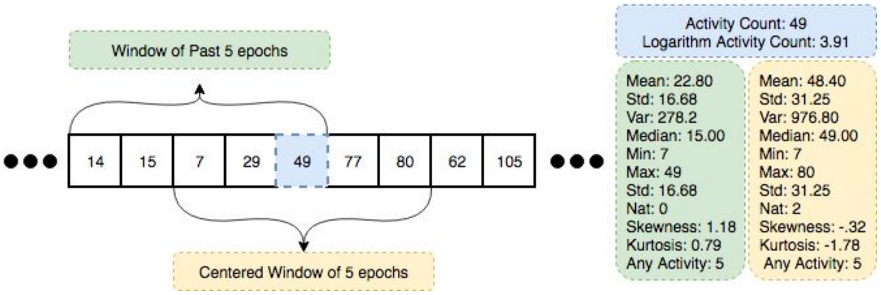

Supplementary Figure 3: Example of calculated features with a window size of 5. Features are calculated using both centered and non-centered (past) windows.

Hyperparameters Tuning for Standard Machine Learning Algorithms

The performance of machine learning methods are influenced by the choices of hyperparameters. In this work, we used a grid search approach to tune hyperparameters, i.e., we exhaustively combined different values for each hyperparameter in order to find the best combination of them. The best parameter set of each model was selected by picking the values of hyperparameters that yielded the highest accuracy score in a 5-fold cross-validation experiment using the MESA training set.

Next we described, for each ML technique, the hyperparameters that were tuned and their values. In bold, we show the best value for each hyperparameter which was selected and used in the test phase. The implementation of the ML methods used in our work is provided by Scikit-Learn toolkit v19.02[1].

- Logistic Regression[2]: loss (**log**), penalty (**l1**, l2, elasticnet), fit intercept (True, **False**), max iter (5, 10, **20**), class weight (**None**, balanced), warm start (False, **True**), alpha (10^{-1}, 10^{-2}, 10^{-3}, $\mathbf{10^{-4}}$, 10^{-5}, 10^{-6}).
- Perceptron[2]: loss (**perceptron**), penalty (l1, l2, **elasticnet**), fit intercept (True, **False**), max iter (10, 10^2, $\mathbf{10^3}$), class weight (None, **balanced**), warm start (False, **True**), alpha (10^{-1}, $\mathbf{10^{-2}}$, 10^{-3}, 10^{-4}, 10^{-5}, 10^{-6}).
- Linear SVM[2]: loss (**hinge**), penalty (l1, l2, **elasticnet**), fit intercept (True, **False**), max iter (5, **10**, 20), class weight (None, **balanced**), warm start (**False**, True), alpha (10^{-1}, 10^{-2}, $\mathbf{10^{-3}}$, 10^{-4}, 10^{-5}, 10^{-6}).
- Extra Trees[3]: number of estimators (8, 64, 128, **512**, 1024), criterion (gini, **entropy**), class weight: (**None**, balanced, balanced subsample), max depth (None, 10, **20**), min impurity decrease (**0.0**, 0.1,

[1]https://scikit-learn.org/0.19/documentation.html
[2]https://scikit-learn.org/0.19/modules/generated/sklearn.linear_model.SGDClassifier.html
[3]https://scikit-learn.org/0.19/modules/generated/sklearn.ensemble.ExtraTreesClassifier.html

0.2).

Evaluation Metrics

Supplementary Table 3: Confusion matrix

		**Actual PSG class (Ground Truth)**	
		Sleep	Wake
**Predicted Class**	Sleep	True Pos. (TP)	False Neg. (FN)
	Wake	False Pos. (FP)	True Neg. (TN)

The following metrics, calculated per participant and averaged over all participants, can be defined based on the confusion matrix of Supplementary Table 3:

- **Accuracy** is the proportion of correctly classified epochs:

$$Acc = \frac{TP + TN}{TP + TN + FN + FP}$$

- **Sensitivity**, also known as **recall** or **true positive rate**, is the proportion of actual sleep epochs that are correctly identified as such:

$$Sen = \frac{TP}{TP + FN}$$

- **Specificity** is the proportion of actual wake that are correctly identified as such:

$$Spe = \frac{TN}{TN + FP}$$

- **Precision**, also known as the **positive predictive value**, is the proportion of actual sleep among all epochs *predicted* as such:

$$Pre = \frac{TP}{TP + FP}$$

- $\mathbf{F_1}$ **score** aims to balance precision and sensitivity. Formally, it is defined as the harmonic mean of both precision and sensitivity:

$$F_1 = \frac{2}{\frac{1}{Pre} + \frac{1}{Sen}} = \frac{2 \times Pre \times Sen}{Pre + Sen} = \frac{2 \times TP}{2 \times TP + FN + FP}$$

- Area under the receiver operating curve (**AUCROC**): The receiver operating curve bi-dimensional space is defined by the false positive rate (1 - specificity) and true positive rate (sensitivity), respectively, as x and y axes. The curve depicts the relative trade-off between true positive (benefits) and false positive (costs). Here we calculate the total area under this curve.
- Area under the precision-sensitivity curves (**AUPS**): similarly to AUCROC, we calculate the area under the precision and sensitivity curve. The intuition is depicting the relative trade-off between precision (the actual sleep epochs predicted as such) at different levels of sensitivity.

Supplementary Results

ROC Plots

Supplementary Figures 4a and 4c represent the area under the receiver operating curve (AUROC) for various traditional sleep-wake scoring algorithms, and compare the same with several ML techniques. Supplementary Figures 4b and 4d correspond to the area under the precision-sensitivity curves (AUPS).

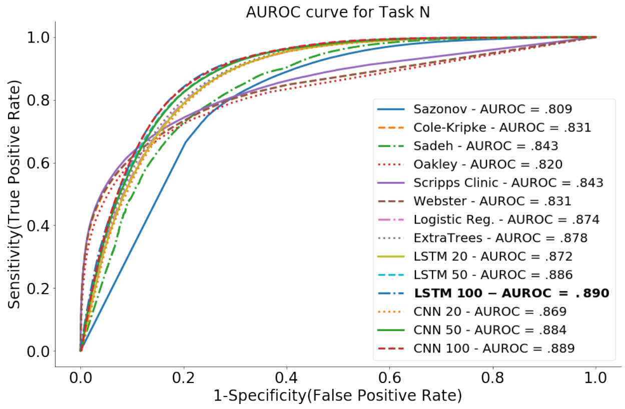

(a)

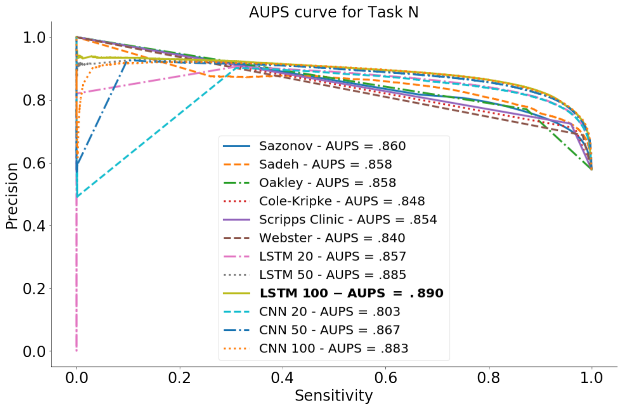

(b)

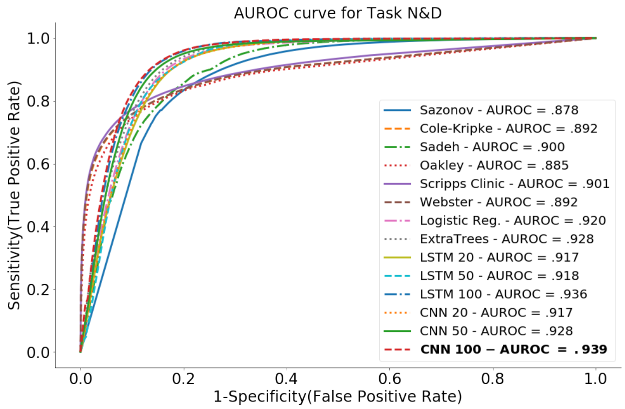

(c)

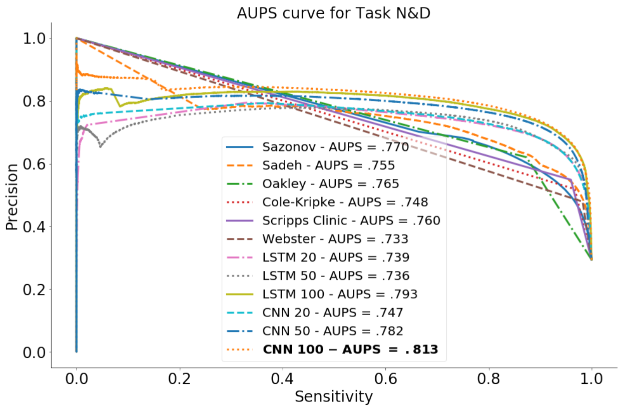

(d)

Supplementary Figure 4: From the above subfigures, we can observe that most of the traditional methods are outperformed by state-of-the-art ML techniques for both Task Night and Task Night&Day with respect to AUROC and AUPS metrics. In particular, the LSTM 100 architecture outperforms all the methods for Task Night achieving 0.890 for both AUROC and AUPS. However, for Task Night&Day, the CNN 100 is superior to other competing methods obtaining an AUROC of 0.939 and an AUPS of 0.813.

Demographic and Clinical Features

Results of the machine learning and deep learning algorithms using extra features for Task Night are shown in Supplementary Table 4. Feature importance of the top 50 features as reported by the Extra Tree algorithm[18] are shown in Supplementary Figure 5. Demographic and clinical features are not among the top 50 features, showing that work still need to be done to include these features in ML models. Features are described below, following the same nomenclature as in MESA documentation:[19]

1. *gender1*: binary variable for gender (0:female, 1: male);
2. *sleepage5c:* participant age;
3. *insmnia5:* binary variable which has value of 1 if participant was told by a doctor as having insomnia, 0 otherwise;
4. *rstlesslgs5:* binary variable which has value of 1 if participant was told by a doctor as having rest less syndrome, 0 otherwise;
5. *slpapnea5:* binary variable which has value of 1 if participant was told by a doctor as having sleep apnea, 0 otherwise.

Supplementary Table 4: Results (Mean ± 95% Confidence Interval) for Task Night. Methods within each group are sorted by their mean accuracy score. Highest/Best results for each category are marked in bold.

Method	Algorithm Evaluation Metrics					Clinical Evaluation Metrics			
	Accuracy	Specificity	Precision	Sensitivity	F1	WASO (min)	MAE WASO	Sleep Eff. (%)	MAE SE
**Machine Learning Algorithms**									
ExtraTrees	81.76 ± 1.03	68.05 ± 1.92	80.25 ± 1.27	90.44 ± 1.18	84.27 ± 1.08	85.24 ± 7.35	42.65	65.79 ± 1.39	10.26
Logistic Regression	81.53 ± 1.04	67.15 ± 1.97	79.97 ± 1.28	90.70 ± 1.20	84.14 ± 1.08	83.79 ± 7.55	44.65	66.31 ± 1.44	10.98
Linear SVM	81.42 ± 1.07	68.09 ± 1.96	80.27 ± 1.28	89.88 ± 1.32	83.83 ± 1.15	87.77 ± 7.87	45.37	65.44 ± 1.47	10.75
Perceptron	79.60 ± 1.14	66.03 ± 2.17	79.54 ± 1.29	87.56 ± 1.70	81.78 ± 1.36	94.70 ± 9.87	53.56	64.88 ± 1.81	12.65
**Rescoring rules applied to Machine Learning Algorithms**									
Resc. Log. Regression	78.88 ± 1.15	80.81 ± 1.78	85.70 ± 1.23	75.80 ± 1.85	78.83 ± 1.48	153.63 ± 10.44	64.71	52.14 ± 1.71	10.65
Resc. ExtraTrees	78.44 ± 1.16	82.05 ± 1.68	86.08 ± 1.24	74.14 ± 1.87	78.14 ± 1.53	160.66 ± 10.27	68.67	50.71 ± 1.66	10.99
Resc. Linear SVM	78.13 ± 1.20	81.51 ± 1.74	85.88 ± 1.23	73.99 ± 1.96	77.67 ± 1.61	161.14 ± 10.69	70.55	50.79 ± 1.74	11.32
Resc. Perceptron	76.63 ± 1.34	79.56 ± 1.95	83.69 ± 1.70	72.23 ± 2.44	74.96 ± 2.00	164.69 ± 12.93	80.24	50.67 ± 2.13	13.80
**Deep Learning Algorithms**									
LSTM 100	83.06 ± 0.98	70.19 ± 1.95	81.75 ± 1.25	91.01 ± 1.08	85.35 ± 1.02	81.88 ± 7.80	41.90	65.28 ± 1.42	9.70
CNN 100	83.06 ± 1.01	70.16 ± 2.01	81.74 ± 1.29	91.11 ± 1.17	85.30 ± 1.07	81.61 ± 7.97	44.24	65.42 ± 1.47	10.17
LSTM 50	82.57 ± 1.00	69.41 ± 1.93	81.22 ± 1.26	90.84 ± 1.07	84.99 ± 1.03	82.79 ± 7.58	41.11	65.49 ± 1.39	9.81
CNN 50	82.35 ± 1.04	67.69 ± 2.03	80.52 ± 1.31	91.74 ± 1.08	84.95 ± 1.06	77.88 ± 7.54	44.18	66.79 ± 1.43	10.83
CNN 20	81.31 ± 1.02	66.37 ± 1.95	79.55 ± 1.29	90.84 ± 1.13	84.03 ± 1.07	81.82 ± 7.28	43.70	66.75 ± 1.39	10.96
LSTM 20	81.40 ± 1.01	68.53 ± 1.90	80.37 ± 1.27	89.40 ± 1.17	83.86 ± 1.07	89.55 ± 7.44	41.08	64.99 ± 1.40	9.88
**Rescoring rules applied to Deep Learning Algorithms**									
Resc. LSTM 100	81.16 ± 1.00	78.54 ± 1.81	85.18 ± 1.23	81.40 ± 1.52	82.09 ± 1.23	127.77 ± 9.56	47.36	56.37 ± 1.57	8.62
Resc. CNN 100	80.43 ± 1.02	79.30 ± 1.80	85.43 ± 1.24	79.55 ± 1.67	80.99 ± 1.31	135.64 ± 10.08	54.04	55.01 ± 1.65	9.58
Resc. CNN 50	80.18 ± 1.05	78.29 ± 1.85	84.77 ± 1.27	79.85 ± 1.64	80.91 ± 1.30	133.86 ± 9.87	52.02	55.64 ± 1.65	9.49
Resc. LSTM 50	80.24 ± 1.05	79.59 ± 1.76	85.46 ± 1.22	79.02 ± 1.59	80.87 ± 1.28	138.26 ± 9.76	53.05	54.53 ± 1.60	9.13
Resc. LSTM 20	77.64 ± 1.14	82.54 ± 1.68	86.34 ± 1.25	72.22 ± 1.88	77.06 ± 1.52	168.52 ± 10.39	74.71	49.41 ± 1.67	11.87
Resc. CNN 20	78.56 ± 1.13	80.80 ± 1.75	85.55 ± 1.27	75.17 ± 1.85	78.45 ± 1.49	155.56 ± 10.33	64.77	51.83 ± 1.68	10.73

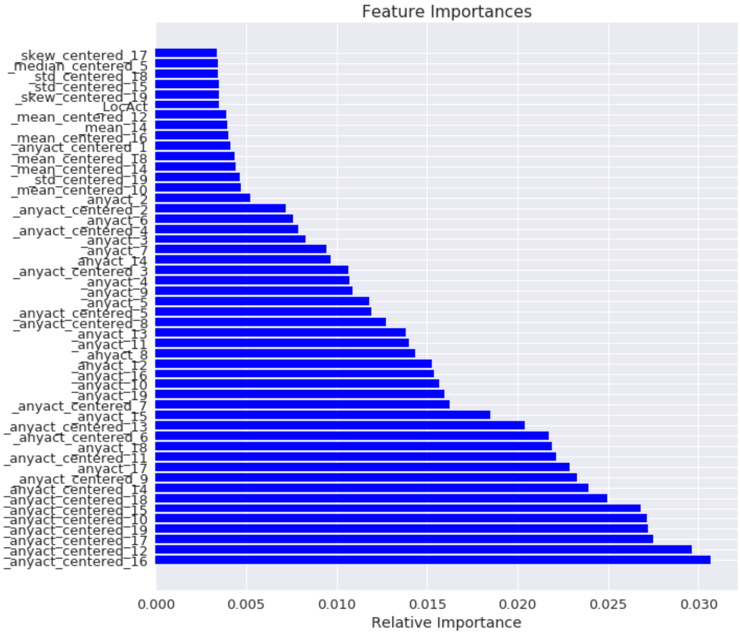

Supplementary Figure 5: The relative important of the best 50 features as calculated by the Extra Trees algorithm. Note that none of the demographic and clinical features are not shown among the top 50.

Supplementary References

[1] Webster, J. B., Kripke, D. F., Messin, S., Mullaney, D. J. & Wyborney, G. An activity-based sleep monitor system for ambulatory use. *Sleep* **5**, 389–399 (1982).

[2] Cole, R. J., Kripke, D. F., Gruen, W., Mullaney, D. J. & Gillin, J. C. Automatic sleep/wake identification from wrist activity. *Sleep* **15**, 461–469 (1992).

[3] Sadeh, A., Sharkey, M. & Carskadon, M. A. Activity-based sleep-wake identification: an empirical test

of methodological issues. *Sleep* **17**, 201–207 (1994).

[4] Oakley, N. Validation with polysomnography of the sleepwatch sleep/wake scoring algorithm used by the actiwatch activity monitoring system. *Bend: Mini Mitter, Cambridge Neurotechnology* (1997).

[5] Sazonov, E. *et al.* Activity-based sleep–wake identification in infants. *Physiological measurement* **25**, 1291 (2004).

[6] Kripke, D. F. *et al.* Wrist actigraphic scoring for sleep laboratory patients: algorithm development. *Journal of sleep research* **19**, 612–619 (2010).

[7] Tonetti, L., Pasquini, F., Fabbri, M., Belluzzi, M. & Natale, V. Comparison of two different actigraphs with polysomnography in healthy young subjects. *Chronobiology international* **25**, 145–153 (2008).

[8] Tilmanne, J., Urbain, J., Kothare, M. V., Wouwer, A. V. & Kothare, S. V. Algorithms for sleep–wake identification using actigraphy: a comparative study and new results. *Journal of sleep research* **18**, 85–98 (2009).

[9] Granovsky, L., Shalev, G., Yacovzada, N., Frank, Y. & Fine, S. Actigraphy-based sleep/wake pattern detection using convolutional neural networks. *arXiv preprint arXiv:1802.07945* (2018).

[10] Kushida, C. A. *et al.* Comparison of actigraphic, polysomnographic, and subjective assessment of sleep parameters in sleep-disordered patients. *Sleep medicine* **2**, 389–396 (2001).

[11] Jean-Louis, G., Kripke, D. F., Mason, W. J., Elliott, J. A. & Youngstedt, S. D. Sleep estimation from wrist movement quantified by different actigraphic modalities. *Journal of neuroscience methods* **105**, 185–191 (2001).

[12] de Souza, L. *et al.* Further validation of actigraphy for sleep studies. *Sleep* **26**, 81–85 (2003).

[13] Weiss, A. R., Johnson, N. L., Berger, N. A. & Redline, S. Validity of activity-based devices to estimate sleep. *Journal of Clinical Sleep Medicine* **6**, 336–342 (2010).

[14] Tudor-Locke, C., Barreira, T. V., Schuna Jr, J. M., Mire, E. F. & Katzmarzyk, P. T. Fully automated waist-worn accelerometer algorithm for detecting children's sleep-period time separate from 24-h physical activity or sedentary behaviors. *Applied physiology, nutrition, and metabolism* **39**, 53–57 (2013).

[15] Marino, M. *et al.* Measuring sleep: accuracy, sensitivity, and specificity of wrist actigraphy compared to polysomnography. *Sleep* **36**, 1747–1755 (2013).

[16] Patel, S. R. *et al.* Reproducibility of a standardized actigraphy scoring algorithm for sleep in a us hispanic/latino population. *Sleep* **38**, 1497–1503 (2015).

[17] Quante, M. *et al.* Actigraphy-based sleep estimation in adolescents and adults: a comparison with polysomnography using two scoring algorithms. *Nature and science of sleep* **10**, 13 (2018).

[18] Geurts, P., Ernst, D. & Wehenkel, L. Extremely randomized trees. *Machine Learning* **63**, 3–42 (2006).

[19] of Atherosclerosis, M. M.-E. S. MESA Exam 5 - Sleep Data Documentation Guide. Tech. Rep. (2014). Report available at `https://sleepdata.org/datasets/mesa/files/m/browser/documentation/MESA_Sleep_Data_Documentation_Guide.pdf`. Accessed on March 24, 2019.
